# Auditory Cortical Maturation in a Child with Cochlear Implant: Analysis of Electrophysiological and Behavioral Measures

**DOI:** 10.1155/2015/890508

**Published:** 2015-12-31

**Authors:** Liliane Aparecida Fagundes Silva, Maria Inês Vieira Couto, Robinson Koji Tsuji, Ricardo Ferreira Bento, Ana Claudia Martinho de Carvalho, Carla Gentile Matas

**Affiliations:** ^1^Departamento de Fisioterapia, Fonoaudiologia e Terapia Ocupacional, Faculdade de Medicina, Universidade de São Paulo, 05360-160 São Paulo, SP, Brazil; ^2^Departamento de Otorrinolaringologia, Hospital das Clínicas, Faculdade de Medicina, Universidade de São Paulo, 05360-160 São Paulo, SP, Brazil

## Abstract

The purpose of this study was to longitudinally assess the behavioral and electrophysiological hearing changes of a girl inserted in a CI program, who had bilateral profound sensorineural hearing loss and underwent surgery of cochlear implantation with electrode activation at 21 months of age. She was evaluated using the P1 component of Long Latency Auditory Evoked Potential (LLAEP); speech perception tests of the Glendonald Auditory Screening Procedure (GASP); Infant Toddler Meaningful Auditory Integration Scale (IT-MAIS); and Meaningful Use of Speech Scales (MUSS). The study was conducted prior to activation and after three, nine, and 18 months of cochlear implant activation. The results of the LLAEP were compared with data from a hearing child matched by gender and chronological age. The results of the LLAEP of the child with cochlear implant showed gradual decrease in latency of the P1 component after auditory stimulation (172 ms–134 ms). In the GASP, IT-MAIS, and MUSS, gradual development of listening skills and oral language was observed. The values of the LLAEP of the hearing child were expected for chronological age (132 ms–128 ms). The use of different clinical instruments allow a better understanding of the auditory habilitation and rehabilitation process via CI.

## 1. Introduction

The central auditory nervous system starts its development in intrauterine life; however, its maturation persists lifelong. The first phase of development is independent of external neurosensory stimulation. However, the second phase will only be effective from the sensory inputs that will organize and direct the process of connections of neural networks development [1]. It is through sound stimulation that cortical maturation is achieved, thanks to a phenomenon called neural plasticity: the ability to be modified in order to improve the cortical response front to environmental stimuli. Through these morphological (axon, dendritic, and synaptic structures) and functional (neuronal and synaptic physiology) changes, memory acquisition and subsequent learning become possible, reflecting behavioral changes with the development of auditory and language skills [2–4].

In cases where there is deprivation of sound stimulation, direct stimulation of the auditory nerve fibers through the cochlear implant (CI) has been an alternative for the CANS to receive the stimulation needed for the maturation process and, consequently, the development of auditory and oral language skills [5, 6]. Hence, after the surgical procedure and activation of CI electrodes, precise speech and language therapy is needed, aiming to monitor the development and maturation of central auditory pathways in order to validate the benefits of the rehabilitation process.

Thus, there are outcome measures, standardized and validated for Brazilian Portuguese, that are able to assess the development of auditory and language skills of this population, providing important information that monitor the rehabilitation process. Concerning objective methods, electrophysiological assessment of hearing, through analysis of morphology and latency of Long Latency Auditory Evoked Potentials (LLAEP), has been a current method to verify the degree of development and the limits of central auditory plasticity. This can be done by monitoring the neurophysiological changes in the population with hearing loss after the onset of hearing intervention with the use of CI [7, 8].

Considering that the development and organization of the central auditory pathways in children are directly related to an effective hearing experience, the P1 wave of LLAEP has been used as a biomarker to evaluate the maturation of the central auditory system in children. These measures, associated with behavioral assessment of auditory and language skills, can assist in verifying the effectiveness of auditory rehabilitation in children with a hearing aid (HA) and/or CI [7, 9–11].

Several studies in the literature suggest that, especially in children implanted early, changes in synaptic connections and synchronization of neuronal transmission occur, reflecting a rapid decrease in latency and a concomitant increase in the amplitude of the LLAEP waves. Some authors observed that these children reached latency values of the P1 component expected for age after three months of implantation [12]. Others have concluded that latency values would reach what is expected for age values after four months of CI use [11] while others have suggested that this would occur between 3 and 6 months [13] and after 6 to 8 months of CI use [9, 14].

Studies combining the results of LLAEP electrophysiological tests with behavioral assessments indicate that the decrease in latency of the P1 component is directly related to the improvement of communicative behaviors (vocalizations) [12], with improvement of speech and language skills [10] and with improvement in speech perception of children [8].

Considering the variability of LLAEP results found in children with CI described in the literature and the scarcity of longitudinal studies on behavioral and electrophysiological changes with the use of the CI, the purpose of this study was to describe auditory maturation and changes on behavioral assessments of a child on early stages of stimulation with CI.

## 2. Case Presentation

This longitudinal case-control study was developed at the Audiology Sector of Department of Physical Therapy, Speech, Language and Hearing and Occupational Therapy and the Discipline/Department of Otolaryngology, Faculty of Medicine, University of São Paulo. The study was approved by the Ethics Committee of CAPPesq-HCFMUSP (process number 0319/11) and the procedures were performed when parents signed the informed consent.

Data from electrophysiological and behavioral hearing assessments was collected at four time points: before CI activation and three, nine, and 18 months thereafter.

LLAEP was recorded with a speech stimulus, syllable/ba/, presented in sound field with speakers positioned at an angle of 90° azimuth, 40 cm away from the side where the child uses the CI. The interstimulus interval was 416 ms and the intensity 70 dBHL, with presentation rate of 1.9 stimuli per second. Band pass filter from one to 30 Hz, gain of 100,000, averaging of 512 stimuli, and response analysis window from −100 ms prestimulus and 500 ms poststimulus were used. Two samples of each subject were collected to verify tracings reproducibility and to exclude doubts regarding waves/artefacts.

The following measures were used for behavioral assessment:

- Infant Toddler Meaningful Auditory Integration Scale (IT-MAIS): the adapted version to Brazilian Portuguese [15] was used. It consists of a structured interview administered to parents of children who have CI. It is composed of 10 questions that assess three aspects of speech perception: vocalization, attention to sounds, and identification of sounds. The maximum score is 40 points.
- Meaningful Use of Speech Scales (MUSS) [16]: the adapted version to Brazilian Portuguese [17] was used. It consists of a structured interview of 10 questions that assesses speech production from the perspective of parents of children using CI. The maximum score is 40 points.
- Glendonald Auditory Screening Procedure (GASP) [18]: the adapted version to Brazilian Portuguese [19] was used. It is an instrument used by audiologists that assesses the speech perception of hearing impaired children. The six tests assess auditory skills from detection to comprehension speech. This battery was administered in a soundproof booth using a clinical audiometer Grason Stadler GSI 61, with sound field system, intensity of 60 dB and speakers positioned at 0° azimuth, and distance of 90 cm from the ear with CI.

The study was conducted with a female child whose diagnosis was performed at six months of age: bilateral profound sensorineural hearing loss of unknown etiology. Hearing aids were fitted bilaterally at one year of age, with limited results for the development of auditory and oral language skills.

The child underwent CI implantation surgery in the right ear, with full insertion of electrodes. The electrodes were activated when the child was 24 months old, when she began speech and language therapy that focused on stimulation of hearing and oral language. After activation, she effectively used the CI, more than 8 hours per day.

Hearing thresholds obtained in the free field assessments in the four time points (Table 1), questionnaire scores (IT-MAIS and MUSS) applied with parents, and the performance in GASP (Table 2) showed changes in the development of auditory and language skills of this child:

- Before activation, the mean of 250 Hz and 500 Hz (85 dBHL) frequencies of her hearing thresholds in free field was below that of the other moments. After activation it was verified that the child answered more consistently to other frequencies (1000 Hz, 2000 Hz, and 4000 Hz). Her hearing thresholds after using the CI gradually decreased: with 3 months of use its mean was 60 dB and with 18 months of use its mean was 38 dBHL.
- From the perspective of the parents, the auditory behavior assessed by IT-MAIS increased from 32.5% at the first to 83% at the last assessment, and language skills, evaluated using the MUSS questionnaire, evolved from 17.5% to 55%, respectively.
- At the assessment of auditory skills preactivation, the child did not detect speech sounds; after 18 months of effective use of CI, she was able to detect all six Ling sounds, recognize her own name, and discriminate vowel sounds.

**Figure 1:**
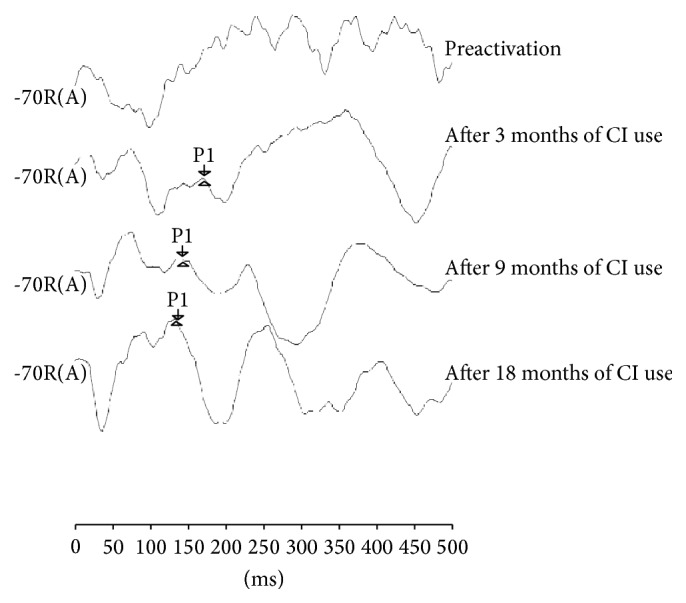
Analysis of LLAEP traces before and three, nine, and eighteen months after activation of electrodes of the CI.

Regarding the electrophysiological assessment of hearing, there was no response in the preactivation period. After three months of electrical stimulation via CI, the presence of the P1 component at 172 ms was observed. These values decreased over the 18 months of CI use, being observed at 141 ms after nine months and 134 ms after 18 months (Figure 1).

**Figure 2:**
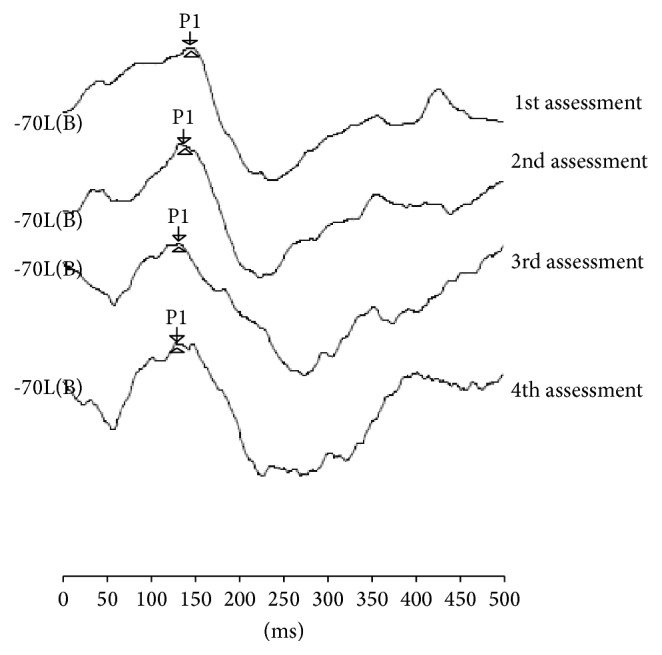
Analysis of LLAEP traces at four assessments of the hearing child.

The results of electrophysiological assessment were compared with the results of a hearing child matched by chronological age and gender, with no hearing or language impairments, whose latency values of the P1 component in the four assessments were, respectively, 132, 132, 130, and 128 ms (Figure 2).

## 3. Discussion

The auditory stimulation enables the nervous system to expand the synaptic connections (neuroplasticity), improving the efficacy of stimulus transmission through the central auditory pathways. This phenomenon is directly related to the development of auditory and oral language skills.

A major difficulty in conducting studies with subjects who are CI users is the heterogeneity of this population, considering that many variables can influence the development of the auditory system and, consequently, the development of auditory and language skills. Among these variables are time of sensory deprivation, age at implantation, age of onset of hearing loss (pre- or postlingual), etiology, speech and language therapy, and family motivation.

For a better understanding of how the maturation of the auditory system occurs via electrical stimulation with the use of CI in children, a longitudinal study with the investigation of auditory changes found in electrophysiological and behavioral assessment of a child with a CI was carried out.

In the initial electrophysiological evaluation of the current study, prior to CI activation, no response was observed. The trace consisted of several positive and negative waves arranged haphazardly, without a reproduction standard even after several recordings. This feature is reported in the literature as, demonstrating the presence of polyphasic waves, the central auditory pathways are abnormally organized front to sound deprivation [8, 20, 21].

In the assessments subsequent to the CI activation, as the auditory experience provided by the effective use of this device increased, the electrophysiological tracings became more defined and latency values of the P1 component gradually decreased. This improvement in electrophysiological findings was possibly observed due to the fact that the peripheral auditory stimulation through the CI was able to provide improved functionality of synaptic connections, which gradually increased the speed of neural transmission and amplification of synaptic synchronization [11, 22].

It was not the aim of this study to evaluate other components of the N1-P2-N2 complex. The literature reports that P1 is the component with the highest occurrence in young children and that the other components will define as maturation occurs; thus, the analysis of these components vary greatly at this age range, making it difficult to compare with what is expected for hearing children [23–26].

When comparing the P1 component of electrophysiological evaluation through LLAEP of the child who is a CI user with that of the hearing child, it was observed that, after 18 months of CI use, the P1 latency (134 ms) component resembled the expected values for chronological age (128 ms). This confirms that, at this age range, the auditory system presents a high degree of plasticity and that the process of auditory rehabilitation was being effective.

While there is no normative data regarding the P1 component latency in the literature, the data obtained in this study is similar to data from other authors. Some authors evaluated the P1 component latency in a group of children who received IC with up to three years and are five months old and observed average values of 378,18 ms at the time of activation and 137,5 ms after 12–18 months of CI use [14]. Other authors also monitored 10 children and observed 313 ms latency values in the preactivation period and 259 and 177 ms after three and six months of CI use [27].

According to the literature, the auditory stimulation of deaf children before 36 months of age allows a very fast maturation of auditory system [8, 28, 29]. After this period, the development occurs differently from that of hearing children [8, 14, 20]. The deeper layers of the cortex undergo maturation processes in the absence of stimulation, and the more superficial layers need stimulation to develop properly [30].

After this critical period, synaptic connection abnormalities occur, resulting in an abnormal neuronal connectivity between neuronal cells, functional disintegration, and immaturity of auditory cortical areas; some auditory areas develop nonauditory functions (visual, somatosensory, etc.) and abnormalities in the restructuring of cognitive functions occur [30].

With respect to behavioral aspects, improvements were observed in hearing thresholds with the CI, which demonstrates that the electrical signal provided by the CI allowed access to the sounds of lower intensity (Table 1). For the aspects related to the development of auditory and oral language skills assessed with MUSS (language from the perspective of parents), IT-MAIS (auditory behavior in the perspective of parents), and GASP (evaluation of the auditory skills), very similar results between the first and second assessment were observed. This occurred possibly due to the fact that a longer use of CI is necessary for the changes of hearing and language to be verified.

According to the estimated curve of normal development [31], the values obtained on IT-MAIS assessment show that this child fits on the lower limit at third and ninth months after CI activation, but her performance is close to the mean on the 18th month (between 88.67% and 95.25%). Therefore, it was possible to note that according to the mother important changes occurred on hearing behavior (variation of 60.5%) and language development (variation of 37.5%) after 18 months of activation and constant use of CI. During this period an audiologist verified an improvement on hearing assessment using GASP. So it is possible to infer that there is an agreement on mother's perception and clinical observation (Table 2).

The results obtained in the electrophysiological and behavioral assessments demonstrated the effectiveness of the habilitation process of a child with a CI. Nevertheless, while the P1 component of the electrophysiological assessment showed rapid changes three months after the intervention and after nine months already approached the expected values for age (141 ms of the child with CI and 130 ms of the hearing child), the development of auditory and language skills required a longer follow-up for changes to be observed and measured. The presence of P1 wave was observed at the third month after activation, and an improvement on hearing and linguistic behavior after nine months after activation was noted (six-month interval between assessments).

Whereas the observed changes in the electrophysiological assessment emerged shortly after the beginning of stimulation, this measure appeared to be a predictor of future behavioral auditory development, thus corroborating with the data presented in the literature that emphasizes the analysis of the P1 component as a biomarker of the development of the auditory system [9, 13, 32].

The use of different clinical instruments allows a better understanding of the auditory habilitation and rehabilitation process via CI. The results of this study allowed the observation that the CI enabled the stimulation of auditory system structures in order to foster the development of auditory and oral language skills.

Thus, the combined results of the electrophysiological and behavioral assessment analysis seems to be a proposal for monitoring the results obtained after the intervention via CI to provide information on the results of CI and to guide the therapeutic process [9, 11, 13, 30, 33, 34]. Longitudinal studies combining various procedures and with a larger number of subjects are needed to guide future directions and proposals.

## Figures and Tables

**Table 1:** Hearing thresholds in free field of the child with hearing loss with the electronic device.

Length of stimulation	Intensity/frequencies					
	250 Hz	500 Hz	1000 Hz	2000 Hz	4000 Hz	Média
	(dBHL)	(dBHL)	(dBHL)	(dBHL)	(dBHL)	(dBHL)
Preactivation (with HA)	75	95	NT	NT	NT	85
3 months of CI use	55	65	50	70	NT	60
9 months of CI use	45	50	40	55	70	52
18 months of CI use	45	40	30	30	45	38

*Note*. HA: hearing aid; CI: cochlear implant; NT: not tested.

**Table 2:** Behavioral results at the four assessments.

Assessment	Preactivation (with HA)	3 months of CI use	9 months of CI use	18 months of CI use
IT-MAIS	32,5%	22,5%	45%	83%
MUSS	17,5%	17,5%	32,5%	55%
GASP	Did not detect speech sounds	Did not detect speech sounds	Detected all six Ling sounds; recognized own name	Discriminated vowel sounds

*Note*. HA: hearing aid; CI: cochlear implant; NT: not tested.
